# Eosinophilic Esophagitis: Review and Update

**DOI:** 10.3389/fmed.2018.00247

**Published:** 2018-10-09

**Authors:** Elisa Gomez Torrijos, Rosario Gonzalez-Mendiola, Manuela Alvarado, Robledo Avila, Alicia Prieto-Garcia, Teresa Valbuena, Jesus Borja, Sonsoles Infante, M. Pilar Lopez, Eva Marchan, Patricia Prieto, Mar Moro, Ana Rosado, Vanessa Saiz, M. Luisa Somoza, Olga Uriel, Angelina Vazquez, Pilar Mur, Paloma Poza-Guedes, Joan Bartra

**Affiliations:** ^1^Hospital General Universitario de Ciudad Real, Ciudad Real, Spain; ^2^Hospital Central de la Cruz Roja San José y Santa Adela, Madrid, Spain; ^3^Hospital San Pedro de Alcántara, Cáceres, Spain; ^4^Hospital Universitario Virgen del Rocío, Seville, Spain; ^5^Hospital General Universitario Gregorio Marañón, Madrid, Spain; ^6^Hospital Infanta Sofía, Madrid, Spain; ^7^Hospital Clinico Universitario Virgen de la Arrixaca, Murcia, Spain; ^8^Hospital Virgen del Valle, Toledo, Spain; ^9^Hospital General Universitario de Albacete, Albacete, Spain; ^10^Hospital Universitario Fundación Alcorcón, Alcorcón, Spain; ^11^Hospital UniversitarioReina Sofía de Córdoba, Cordoba, Spain; ^12^Hospital Universitario Infanta Leonor, Madrid, Spain; ^13^Hospital Universitario de Araba, Vitoria-Gasteiz, Spain; ^14^Hospital Universitario Puerta de Hierro Majadahonda, Madrid, Spain; ^15^Hospital Santa Barbara, Puertollano, Spain; ^16^Allergy Section, Hospital Universitario de La Laguna, San Cristóbal de La Laguna, Spain; ^17^Allergy Section, Pneumology Department, Hospital Clínic Universitat de Barcelona, Barcelona, Spain

**Keywords:** eosinophilic esofagitis, esophagoscopy, eosinophilics, elimination diet, allergens

## Abstract

**Background:** Eosinophilic esophagitis (EoE) was first described in the 1990s, showing an increasing incidence and prevalence since then, being the leading cause of food impaction and the major cause of dysphagia. Probably, in a few years, EoE may no longer be considered a rare disease.

**Methods:** This article discusses new aspects of the pathogenesis, symptoms, diagnosis, and treatment of EoE according to the last published guidelines.

**Results:** The epidemiological studies indicate a multifactorial origin for EoE, where environmental and genetic factors take part. EoE affects both children and adults and it is frequently associated with atopic disease and IgE-mediated food allergies. In patients undergoing oral immunotherapy for desensitization from IgE-mediated food allergy the risk of developing EoE is 2.72%. Barrier dysfunction and T-helper 2 inflammation is considered to be pathogenetically important factors. There are different patterns of clinical presentation varying with age and can be masked by adaptation habits. Besides, symptoms do not usually correlate with histologic disease activity. The diagnostic criteria for EoE has evolved but mainly requires symptoms of esophageal dysfunction with histologic evidence of a peak value of at least 15 eosinophils per high-power field. Endoscopies have to be repeated in order to diagnose, monitor, and treat EoE. Treatment of EoE can be started either by drugs (PPIs and topical corticosteroids) or elimination diets. The multistage step-up elimination diet management approach of EoE is promising. Endoscopic dilation is used for patients with severe dysphagia/food impaction with inadequate response to anti-inflammatory treatment.

**Conclusions:** Research in recent years has contributed to a better understanding of EoE's pathogenesis, genetic background, natural history, allergy workup, standardization in assessment of disease activity, evaluation of minimally invasive diagnostic tools, and new therapeutic approaches. However, several unmet needs are to be solved urgently, as finding a non-invasive disease-monitoring methods and biomarkers for routine practice, the development or new therapies, novel food allergy testing to detect triggering foods, drug, and doses required for initial therapy and safety issues with long-term maintenance therapy, amongst others. Besides, multidisciplinary management units of EoE, involving gastroenterologists, pediatricians, allergists, pathologists, dietitians, and ENT specialists are needed.

## Introduction

There are no eosinophils in healthy esophageal mucosa. During the 1980s, some authors interpreted their presence in the esophageal mucosa as a histological marker of gastroesophageal reflux disease (GERD) (1, 2). Attwood et al. (3) in the USA (1993) and 1 year later Straumann et al. (4) in Switzerland defined eosinophilic esophagitis (EoE) as an entity with its own clinical and histological characteristics. Kelly et al. (5), in 1995, described a series of 23 children with GERD refractory to medical treatment and fundoplication, who responded to treatment with elemental diet. As a result, EoE was recognized as a form of food allergy. The first consensus guide of EoE (6) was published in 2007 and updated in 2011 (7). The American College of Gastroenterology in 2013 and the European and American Societies of Pediatric Gastroenterology (8) in 2014 published their guides for the management of EoE. Finally, the European guide on EoE has also edited some evidence-based statements and recommendations for diagnosis and treatment in children and in adults (9).

## Definition

Currently, EoE is defined as a chronic, local immune-mediated esophageal disease characterized, clinically, by symptoms related to esophageal dysfunction and, histologically, by eosinophil-predominant inflammation (9).

The disease is understood as a clinical-pathological entity, where symptoms and histology must always be considered together, both for the diagnosis and for the follow-up or assessment of the response to treatment (7, 10).

## Epidemiology

In recent years, there has been an increase in the incidence and prevalence of EoE, with an exponential increase in articles published on EoE. In the USA and Europe, EoE is the most frequent cause of dysphagia in children and in young adults (11).

In children undergoing gastroscopy, irrespective of the cause, the prevalence was 3.7%. When gastroscopy was due to impaction or dysphagia, the prevalence was higher: 63–88% in children (12, 13) and 10–15% in adults (14).

In the general population, it is estimated to be about 30–52 cases per 100,000 inhabitants (15). A study conducted in central Spain between 2005 and 2011 (16) showed an average annual incidence of 6.37/100,000 inhabitants and a prevalence of 44.6/100,000 inhabitants, the latter being higher in urban areas (17).

Although EoE has been described in all races and continents, there is a slight predominance among Caucasians. It is more frequent in males than in females (3:1), this difference being more remarked (19:1) in a Spanish study (18). The mean age at the time of diagnosis ranges between 30–50 years in adults (18) and 5.4–9.6 years in children (12).

Some groups have suggested a relationship between EoE and environmental exposure during the pollen season (19). However, a recent meta-analysis has concluded that no significant seasonal variation in the number of patients diagnosed nor in the number of episodes of food impaction that require medical assistance could be observed (20).

## Etiopathogenesis, pathophysiology, and genetics

Eosinophilic esophagitis is regarded as an esophageal inflammatory disease associated with atopic diseases. The genetic variation of thymic stromal lymphopoietin (*TSLP*) and calpain 14 (*CAPN14*) contributes to EoE, but the answer to how this relates to atopy remains unclear. Susceptibility to EoE is mediated by multiple genes, which have synergistic effects. These genes include those of general atopic disease and those specific to EoE (21).

Eosinophilic esophagitis is caused by an adaptive immune response to patient-specific antigens, primarily foods. Whether EoE is IgE-mediated or not is unknown. The most likely outcome is the presence of Th2 lymphocytes with an altered esophageal barrier function. The key cytokines and chemokines involved in the recruitment and remodeling of eosinophils are thymic stromal lymphopoietin, interleukin-13 (IL-13), CCL26/eotaxin-3, and transforming growth factor-β (TGF-β). Chronic food dysphagia, food impaction, and the dreaded late complications are partly related to dense subepithelial fibrosis, probably induced by IL-13 and TGF-β (22).

The deterioration of the epithelial barrier takes place because of genetic predisposition, reflux, and food intake. Invasive allergens and microbial antigens activate the innate and acquired immune systems. Eosinophils degranulate, release toxic proteins, and generate alterations in extracellular DNA, which serve as a defense system but also cause tissue damage. While releasing different cytokines, eosinophils modulate the inflammation and promote its chronification, finally resulting in fibrosis, which, in turn, has negative effects on the barrier function of the skin (23).

The epidemiological studies carried out indicate a multifactorial origin for EoE, where environmental and genetic factors play a role (3). Epidemiological data support the predominance of the disease in men (7, 15, 24) of white race (25), as well as the family aggregation (26) observed in studies with families and in concordance studies between twins (27).

The hygiene hypothesis attributes the rise in EoE to modern hygienic conditions that have resulted in fewer childhood infections with microbes that might have protected against allergy development. Microbial dysbiosis, a change in the microbiome's composition and diversity caused by a modern affluent lifestyle, also might contribute to allergic conditions. Environmental factors that include modern chemicals contaminating crops, livestock treated with hormones and antibiotics, food additives and processing changes, and pollutants in the air and water conceivably might predispose people to EoE. An intriguing hypothesis attributes the increase in EoE to the increase in the use of acid-suppressive medications like proton pump inhibitors (PPIs), which might prevent peptic digestion of food allergens, increase gastric permeability, and alter the microbiome to favor food allergy development. In a recent pediatric case-control study, use of acid suppressants in infancy was by far the strongest risk factor identified for the development of EoE in the later stages of life (28). Specific Ig4 (sIgG4) antibiodies to cow's milk (CM) proteins are common, and a high titer of antibodies can be seen in children with EoE. Although it is unclear whether this response is pathogenic, sIgG4 levels imply that these antibodies are an important feature of the local immune response that gives rise to EoE (29).

A history of esophageal symptoms is found in 10% of the parents of these patients. In addition, if there is an affected sibling, the risk of suffering from a similar disease increases by 80%.

Therefore, it is a complex disease, which does not follow the classic Mendelian pattern, producing interaction of multiple genes; some with a protective effect, others as a risk factor for the disease and whose expression could be conditioned by environmental factors (27).

1. The first genetic variant described was *CCL26* (a gene encoding eotaxin 3 on chromosome 7q11) and its CCR3 receptor. The single nucleotide polymorphism (SNP) of *CCL26* (rs2302009) is associated with the risk of suffering from the disease (30). Six years ago, they described polymorphisms in the promoter region of *TGF-*β*1* (chromosome 19q13). More than *CC509T*, the C allele is associated with a positive response to treatment with corticosteroids and with an increase in its expression in esophageal cells. The TT genotype could even be considered a pro-fibrosis risk factor. In children, a relationship has been found between the variants of the *TGF-*β*1* gene and the degree of severity of EoE in patients sensitized to food (31).

The epidermal differentiation complex (EDC) binds a significant number of structuraly, functionally and evolutionarily related genes that play an important role in the terminal diferentiation of the hunman epidermis. It is located within the region q21 in the human chromosome 1. The alterations that produce is what is known as the transcriptome of eosinophilic esophagitis. Whitin this chromosome are encoded several genes (fillaggrin, invlucrin, SSPRR family…) that intervene in the epithelial esophagus barrier. Therefore, the EDC is not a gene, it is a set of genes all od them encoded in human chromosome 1, in the 1q21 region (32). This set of alterations is known as the transcriptome of the EoE, which allows us to distinguish patients with EoE from healthy controls. Reversibility has been demonstrated after the remission of the disease following treatment with corticosteroids. Single nucleotide polymorphisms could be seen in the following cases.

Filaggrin (2288del4): whose loss of function is associated with alterations in the esophageal barrier, increasing permeability and susceptibility to allergens (32);

Desmoglein1 (transmembrane desmosomes): determines loss of barrier function and increases levels of IL-5 and TLSP;

Periostin (POSTN) (adhesion molecule that regulates the deposition of the extracellular matrix): an increase in its expression causes an increment in the process of esophageal remodeling.

1. Genome-wide association studies (GWAS)

They were first published in 2010. Single nucleotide polymorphism was found in the gene that codes for TSLP and WDR36 (chromosome 5q22) (33), whose expression increased in EoE.

Likewise, genes related to cellular autophagy (*ATG7*) are detected more frequently in active EoE, contrary to what occurs in EoE in remission, in GERD, or in normal subjects. So, it could be considered as a biomarker of EoE (34).

Recently, it has been investigated that the toll-like receptor 3 (*TLR3*) () constitutes a novel genetic susceptibility locus for developing EoE, and the effects would be independent of *TSLP* (35).

## Eosinophilic esophagitis, gastroesophageal reflux disease, and response to proton pump inhibitors

During the 1980s, the presence of eosinophils in the esophageal mucosa was described in patients with GERD (1, 2). Subsequently, EoE was identified as an entity with its own clinical and histological characteristics (3, 4). Both syndromes, GERD and EoE, can overlap and coexist in the same subject (36).

The clinical guidelines of EoE indicated that to reach the diagnosis of EoE, prior treatment with PPIs should be done at a double dose for 8 weeks to rule out GERD (7, 8). Later, it was shown that omeprazole provides an anti-inflammatory effect independent of its anti-acid effect. As a result, the response to PPIs failed to differentiate patients with GERD from patients with EoE (37).

In the updated consensus of 2011, a group of patients with clinical characteristics of EoE and response to PPIs was recognized (7, 38). They were called PPI-responsive esophageal eosinophilia (PPI-REE) patients. Subsequent studies have shown that this group of patients did not differ from EoE patients, neither in the clinical, endoscopic, or histological characteristics nor in the expression of Th2 cytokines and their transcriptome (36, 38–45).

Secretion of Th2 cytokines can be inhibited by PPIs in a manner similar to corticosteroids in EoE (43). Thus, some experts regard that the treatment of EoE would start with PPIs, since they are safe drugs. In the case that eosinophilic inflammation subsided, we would be facing a PPI-REE. This name, according to experts, should be abandoned as it is an inadequate descriptor of this pathology (46, 47). The main novelty of the new guidelines on EoE (9) is the retraction of the term “PPI-REE,” considering PPIs are at the same level as diets or topical steroids in the treatment of EoE and are not a diagnostic criteria anymore.

### Symptoms

Symptoms in EoE are due to esophageal dysfunction, which can appear at any age. Nevertheless, it can be detected more frequently in children and in young adults until the 5th decade of life (18).

Some patients present symptoms constantly, others intermittently, remaining asymptomatic between periods of exacerbation (7). The symptoms can be present for a long time (mean of 3–5 years) before reaching a diagnosis of EoE in children and in adults, especially if the disease appears progressively (7, 48).

However, the diagnosis is not uncommon after a short history or even after an acute episode, mainly after an impaction (49). Clinical presentation varies according to age, evolving from non-specific symptoms such as abdominal pain and rejection of food in pediatric patients to more pronounced esophageal symptoms such as dysphagia in adult patients (50).

In preschoolers, food refusal and intake of lesser amounts than those appropriate for their age are what we find more commonly. All of these along with vomiting, irritability, and abdominal pain will result in weight loss and failure to thrive.

In school children, food refusal, difficulties in introducing new foods in the diet, the preference for liquids and soft diets, and a tendency to be “slow eaters” are to be noted. In this group of children, abdominal pain and vomiting are the most frequent gastrointestinal symptoms.

In older children and adolescents, dysphagia and chocking are the most noteworthy symptoms. Impaction is more common in such cases than at early ages. They also maintain a preference for soft diets and fluid intake, tend to have a little varied diet, and may show fear and anxiety at the time of meals (51, 52).

Sometimes the first symptoms may be diarrhea and/or bloody stools, complicating early diagnosis (53). In many cases, children with EoE present vomiting and abdominal pain, which suggest gastroesophageal reflux disease. But in these cases, the symptoms are refractory to treatment (54).

In adults, dysphagia for solids and impaction of food (50, 55) are the principal symptoms. Chest pain can be associated with adults (50, 55–60). However, some of them only show symptoms of GERD or non-specific pharyngeal discomfort (59). Rarely, there could be spontaneous esophageal perforation due to intense vomiting (Boerhaave syndrome) after food impaction (56).

It has been postulated that these differences in the clinical presentation between pediatric and adult patients could conform to different causes; pediatric patients fail to express their symptoms in the same manner as adults, the time of evolution and the progression of the inflammatory disorder varies from an inflammatory phenotype in chilhood to a fibrostenotic one in adulthood (55).

The symptoms can also be masked, both in children and in adults, because along the years, the patients carry out dietary modifications to avoid the development of symptoms. This is why the medical history should include the following questions: “Do you chew your food a lot?” “Are you the last to get up from the table?” “Do you drink plenty of water during the meal to help you swallow?” “Do you avoid eating certain foods such as bread, rice, or meat?” “Do you cut the food into very small pieces?” (56, 61).

In conclusion, there are different patterns of clinical presentation varying with age, and they can be masked by adaptation habits (56, 61). In addition, the severity of the symptoms does not necessarily correlate with the density of the eosinophilic inflammation (62, 63). On the whole, symptoms alone are not enough to diagnose or to assess the response to treatment.

### Diagnosis

The presence of the following diagnostic criteria is required for the diagnosis: (a) symptoms of esophageal dysfunction; (b) eosinophilic esophageal inflammation, with ≥15 eosinophils per high-power field (eos/hpf), affecting the esophagus alone; and (c) excluding other causes of esophageal eosinophilia (Table 1) (7, 8).

**Table 1:** Other causes of esophageal eosinophilia.

Eosinophilic Gastroenteritis
Celíac Disease
Crohn's disease
Infection
Hypereosinophilic syndrome
Achalasia
Hypersensitivity to drugs
Vasculitis
Pemphigus
Connective tissue diseases

**Figure 1:**
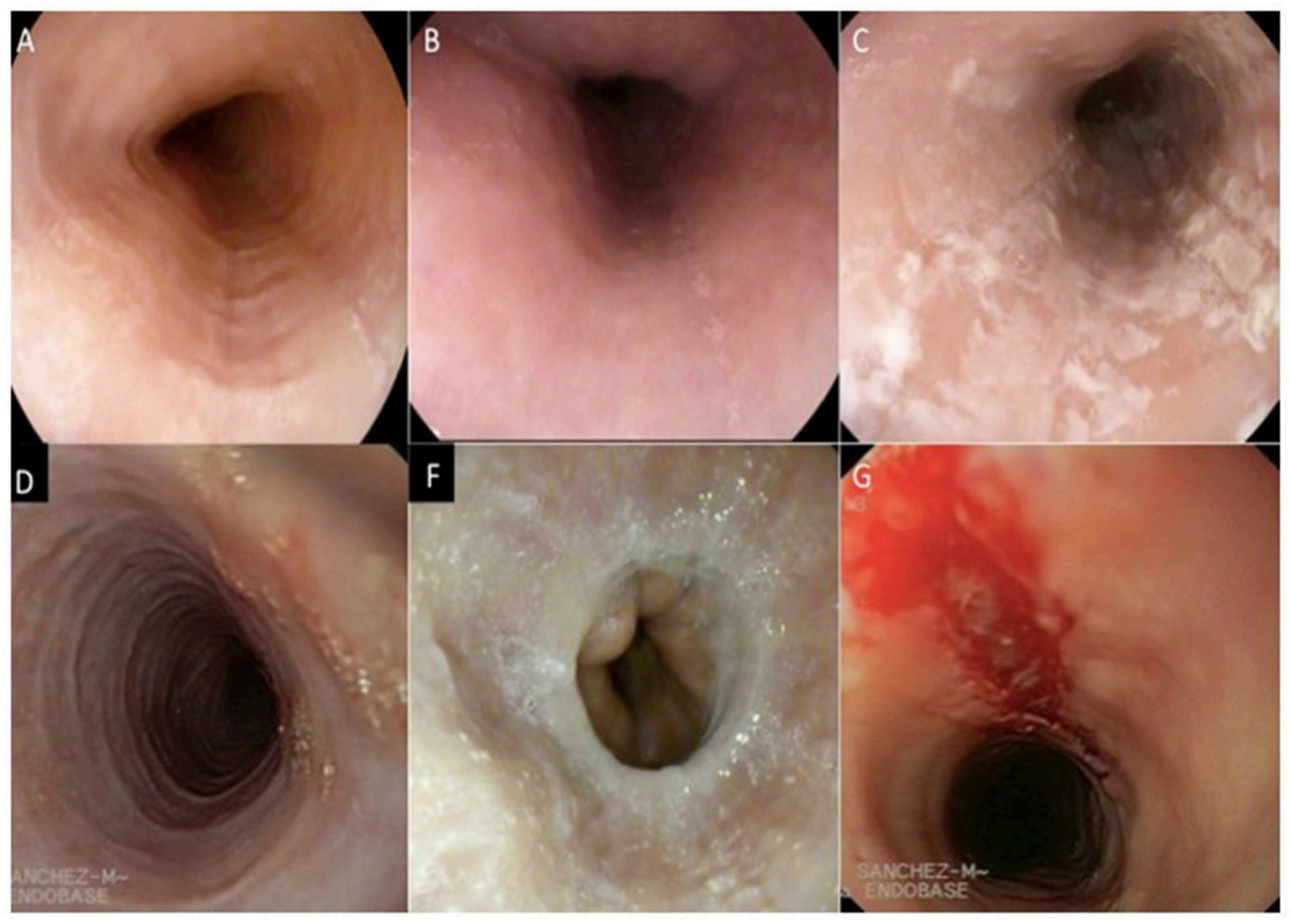
Endoscopic signs of the eosinophilic esophagitis. **(A)** longitudinal furrows, **(B)** Edema, **(C)** Exudate, **(D)** Pseudo rings, **(F)** Stenosis, **(G)** Fragility of the mucosa. (Images provided by the Multidisciplinary Group of Eosinophilic Esophagitis of the General University Hospital of Ciudad Real).

Upper gastrointestinal endoscopy is the first diagnostic test to be performed when EoE is suspected. It includes the inspection of the esophagus, stomach, and duodenum, acquisition of esophageal biopsies of the previous places, and elimination of other pathologies (64). The endoscopic signs of EoE are shown in Table 2, Figure 1.

**Table 2:** Endoscopic findings in eosinophilic esophagitis.

**Signs of inflammation**	**Signs of fibrostenosis**
Longitudinal furrows/ridges	Fixed esophageal rings “trachealization”
Exudates/White spots	Feline esophagus
Pale, edematous mucosa, decreased vascularity	Esophageal rings or diffuse esophageal stenosis, narrow caliber esophagus
Fragile mucosa, crêpe paper esophagus, with lacerations at the passage of the endoscope	

These endoscopic signs are neither pathognomonic of EoE nor are they enough to make diagnostic decisions (65). White exudates can be mistaken for candidiasic esophagitis and taking samples for fungal culture should be considered, mainly in patients treated with topical corticosteroids (TCs). Rings and stenosis are more common in adults than in children (57 and 25%, respectively). Biopsies will always be performed, because up to 10% of the adult patients and 32% of the pediatric patients with a rigorously normal endoscopy pattern present esophageal eosinophilia (66). Recently, a grading system for the severity of endoscopic findings has been developed to evaluate the degree of disease activity (67). This proposed system for endoscopically-identified esophageal features of EoE defines common nomenclature and severity scores for the assessment of EoE disease activity. The system has good interobserver agreement among practicing and academic gastroenterologists (Table 3).

**Table 3:** Endoscopic scoring reference system to improve the reliability of endoscopic findings (67).

1) Major characteristics:
- Rings: (includes trachealization and transient rings) Grade 0: none to Grade 3: severe (which do not allow the passage of the endoscope) - Excluded (includes points and plates): Grade 0: none to Grade 2: severe (more than 10% of the esophageal surface) - Edema (decreased vascularity): Grade 0: absent to Grade 1: present - Furrows: Grade 0: absent to Grade 1: present - Stenosis or contractions: Grade 0: absent to Grade 1: present.
2) Minor features:
- Mucosa in “paper-crepe”: Grade 0: absent to Grade 1: present.

According to the histology of EoE, esophageal eosinophilia is irregular and variable between distal and proximal esophagus. Currently, the recommendation is to take at least six biopsies from two different sites, typically from the distal and proximal esophagus (7, 66). It is advisable to perform a biopsy for those areas with macroscopic inflammatory findings and for antrum and duodenum to rule out eosinophilic gastroenteritis (mainly in children and in adults with gastric or intestinal symptoms). In addition to the number of eosinophils, there are other histopathological findings associated with EoE that should also be reported (Table 4). Since symptoms do not correlate exactly with the activity of the disease, histology is still necessary to control the disease. In clinical practice, it is enough to perform haematoxylin-eosin staining for histological evaluation (9).

**Table 4:** Histological findings in eosinophilic esophagitis.

. ≥15 eosinophils per high-power field
. Disposition of eosinophils in superficial layers of the epithelium
. Disposition of eosinophils in accumulations or microabscesses
. Extracellular eosinophilic granules

Lin et al. developed a new method for analysis and presentation of esophageal distensibility data using high-resolution impedance planimetry recordings during a volume-controlled distention. The results show that the patients with EoE and normal endoscopy had esophageal distensibility parameters similar to those of normal controls, whereas patients with EoE and stricture or narrow caliber had much lower distensibility than patients with EoE and normal endoscopy. The functional luminal imaging probe (FLIP) topography plots provided a global assessment of the esophageal distensibility along the axial plane of measurement that differentiated patients with varying degrees of endoscopic abnormality. So, new techniques can be leveraged to improve data analysis and presentation using EndoFLIP assessment of the esophageal body in EoE, since they may be helpful in defining phenotypes and guiding treatment strategies (68).

Allergological study is recommended yo every patient, since there is a strong association between EoE and atopy. On the one hand, about 50% of the patients present with peripheral blood eosinophilia. On the other hand, elevated levels of total Ig E can be detected in 80% of the patients. The association with rhinitis, asthma, eczema, or IgE-mediated food allergy is detected in 70, 40, 30, and 50% of the cases, respectively (7, 18). More than 80% of the patients have skin tests and/or IgE specific serum tests positive for inhalants or food. We can also find a profile of patients with negative allergy tests to all foods and who also respond to elimination diets.

Therefore, all patients require an evaluation by an allergist (7, 8), for the complete assessment of their allergic pathology as a whole. In addition, what is more important is that a correct interpretation of allergy tests is needed for avoiding unnecessary or undesirable food restrictions. The following analyses are recommended: hemogram (to detect peripheral blood eosinophilia) and general biochemistry including vitamins and micronutrients to assess the nutritional status. Skin prick test (SPT) should be performed with aeroallergens and with the group of six foods (milk, wheat, soybean/legumes, egg, peanut/nuts, and fish/shellfish) that are most frequently described as EoE-causing. Measures of total IgE and food specific IgE are also useful for the assessment of other associated pathologies, based on the clinical history of each patient.

Although SPT might be helpful in the design of elimination diets in about 50% of the patients, it is not enough to identify the offending foods that cause EoE.

The measurement of biomarkers in peripheral blood has not been helpful until now. Total IgE, eosinophil cationic protein (ECP), eosinophil-derived neurotoxin, tryptase, numerous cytokines, and a fraction of exhaled nitric oxide have been studied (69–71). Only peripheral blood eosinophils have proven to have a significant correlation with the degree of esophageal eosinophilia and its descent, after treatment with corticosteroids or PPIs, though their accuracy for diagnosis and their evaluation of disease activity are suboptimal (9).

The leukotriene C4 (*LTC4*) S mRNA and *TSLP* mRNA are elevated in a subgroup of the patients, and this could help in the differentiation between patients with EoE and an allergic-type phenotype (65).

Minimally invasive diagnostic tests such as esophageal string test and cytosponge have indicated good preliminary correlation with the degree of esophageal eosinophilia and eosinophilic derived proteins, but these data should be corroborated with larger studies (9, 72).

Recently, several promising minimally invasive biomarkers for EoE have emerged; however, only few of these are able to distinguish EoE from other atopic diseases (73).

## Eosinophilic esophagitis and IgE-mediated allergy

Patients with EoE usually suffer from a high number of concomitant atopic disorders that include rhinitis, asthma, and eczema. Besides, IgE-mediated food allergies are common in EoE patients.

The risk of developing EoE in patients undergoing oral immunotherapy for desensitization from IgE-mediated food allergy is 2.72% (74). There are cases described in the literature where patients showing allergic reactions to peanut (75), milk (76, 77), and egg (78, 79) develop EoE.

There are cases of patients who developed EoE after being treated with sublingual immunotherapy with pollen and mites that remitted with the withdrawal of immunotherapy (80, 81).

An occupational aeroallergen can also trigger EoE. There are two cases described. A female cook with bird-egg syndrome and occupational asthma due to egg manipulation developed EoE. The patient suffered an EoE reactivation when she ate poultry meat and by inhalation when she handled egg yolk (82). The other patient was a female baker who developed asthma and EoE by inhalation after exposure to wheat flour, but she was tolerant to wheat when exposed via the digestive route (83).

## Eosinophilic esophagitis: differential diagnosis

Esophageal eosinophilia is not an exclusively anatomopathological finding of EoE. Therefore, a differential diagnosis must be made with respect to other digestive and extradigestive pathologies (Table 1) (8).

**Figure 2:**
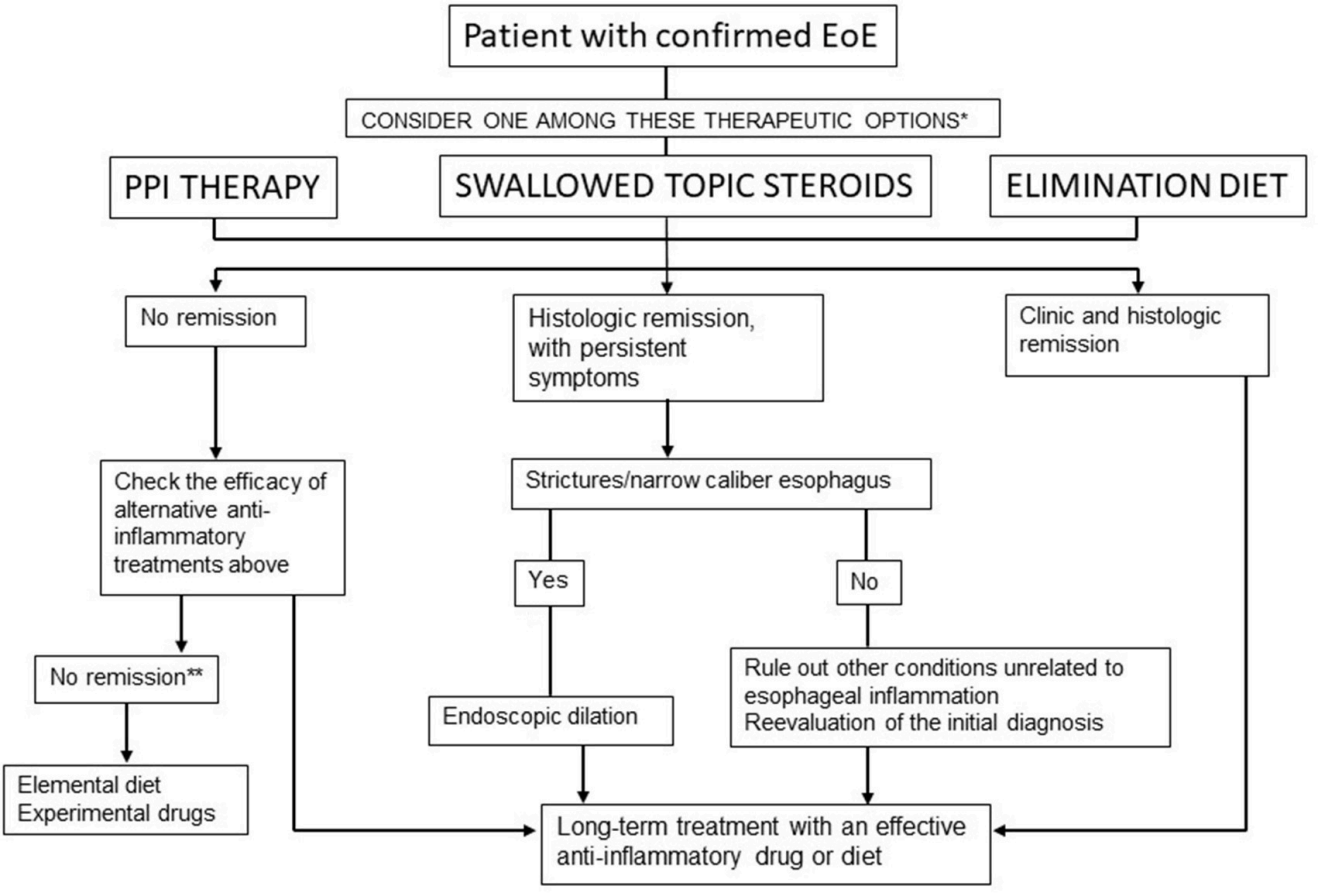
Therapeutic algorithm for EoE in clinical practice. Lucendo et al. (9). *In patients with persistent symptoms under anti-inflammatory therapy, endoscopic dilation should be considered. **Refer the patient to an EoE center.

According to the latest guidelines (9), GERD and PPI-REE (currently PPI-responsive EE) are no longer considered in the differential diagnosis. Eosinophilic esophagitis and GERD can coexist in the same patient; therefore, they are not exclusive pathologies. In addition, PPI-REE (responsive EoE) and EoE have similar phenotypic, molecular, and pathophysiological characteristics and response to the treatment. With regard to this, they are not classified as different pathologies. The response to PPIs is not used for differential diagnosis but rather as a treatment for EoE (84, 85) (Figure 2).

## Natural history and prognosis

In EoE, the risk of developing esophageal stenosis increases with age and the delay in diagnosis and treatment (48, 86–88). Two of the most relevant studies on the progression of EoE in adults were conducted in a single center in Switzerland, where they demonstrated the persistence of dysphagia and eosinophilic inflammation, mainly in children, evolving into subepithelial fibrosis in adults (89, 90). Clinically and histologically active EoE must be treated. An EoE therapy should achieve two therapeutic goals: the first goal is to resolve symptoms and to improve, therefore, the quality of life; and the second goal is to control inflammation, so as to prevent esophageal damage caused by tissue remodeling. For children, the primary goal is to ensure normal growth and development.

Eosinophilic esophagitis is a chronic disease; as such, a long-term follow-up and therapeutic strategy is needed. An analogy of EoE has been made with bronchial asthma, since both are chronic pathologies and include tissue remodeling associated with Th2 cells.

Eosinophilic esophagitis is not life threatening; nevertheless, if left untreated it may cause permanent damage to the esophagus. However, as a chronic disease, the quality of life is substantially impaired as long as EoE is not properly treated. In children (7), modifications of eating habits to avoid dysphagia (see Diagnosis) also affect their social and psychological behavior (anxiety, depression, school problems, etc). The most frequent complications are as follows: esophageal stenosis, feeding impaction, esophageal perforation, and malnutrition. There is no evidence that EoE is a premalignant condition. No case of progression toward esophageal carcinoma or hypereosinophilic syndrome has been described. Nevertheless, an association between EoE and granular cell esophageal tumors has been reported recently (91).

In conclusion, there is an increasing incidence and prevalence of EoE. The considerable delay between symptom onset and diagnosis suggests that there are still clinicians who fail to recognize the disease, which may have important clinical implications (92).

### Treatment

The three basic pillars in the treatment of EoE are as follows: drugs, elimination diets, and endoscopic dilation. The first two act on inflammation, and the third one acts on established fibrosis.

Treatment of EoE could be started by any one of the two options, either drugs or elimination diets. The first step in the treatment could be constituted by PPIs as these drugs are quite safe. In patients who fail to respond to PPIs, the second step could be elimination diets or TCs. Treatment must be individualized according to each patient or their family concerns and lifestyles, and it might be interchangeable over time (46).

The long-term therapeutic strategies and best maintenance doses for pharmacological therapies are not well-defined yet. A reasonable approach is to decrease the dose to the lowest level that maintains the clinical and histological remission until more data are available.

A) Pharmacological treatment (Table 5)

**Table 5:** Drugs used in the treatment of eosinophilic esophagitis.

- Proton-pump inhibitor
Omeprazole and Esomeprazole^*^ • Children from 10 to 20 kg: 10 mg twice a day • Children >20 kg: 20 mg twice a day • Adults: 40 mg twice a day Initial dose 8 weeks. After minimum effective maintenance dose ^*^ It should not be used in children under 12 years of age
Swallowed topical corticosteroids
Fluticasone • Children: 88 to 440 μg twice a day • Adults: 440 to 880 μg twice a day Budesonide - Maintenance: minimum effective dose - Children: 0.5 mg twice a day - Adults: 1 mg twice a day - Maintenance: minimum effective dose
Oral corticosteroids
- Only in serious cases: Prednisone 1–2 mg /kg/day

A-1) Proton pump inhibitors: a recent systematic review with meta-analysis detected that they induce histological remission in at least 50% of the patients and clinical remission in an even higher percentage of patients (50, 93). The reason why some patients are responders and others are not has to be investigated.

In a retrospective study in adults and a prospective study in children, with a follow-up meta-analysis of 73 and 78.6% of the patients, respectively, the adults and children maintained remission with a minimum dose of PPI (94, 95). Patients with reactivation obtained remission by increasing the dose of IBPs to twice daily.

After achieving a remission, the patient should be put on the lowest PPI dose for maintenance treatment (Figure 2).

A-2) Topical corticosteroids: several randomized studies have shown the efficacy of TCs against placebo, improving symptoms and reducing eosinophilic esophageal inflammation. There are different percentages of response depending on the drug, dose, or formulation used (63, 96–101). Eosinophilic inflammation recurs when treatment is withdrawn (7, 8, 102). There is no formulation that has been specifically approved for EoE, hence devices designed for asthma or rhinitis are used. Topical corticosteroids have to be swallowed twice daily and the patient should not eat or drink for the next 30–60 min.

The most commonly used TCs are fluticasone propionate and budesonide. They can be sprayed using a metered dose inhaler (MDI) device and then swallowed (96, 103); a liquid formulation for intranasal administration or nebulization and a viscous oral suspension prepared with sucralose that can be swallowed directly are also available (100). Moreover, ciclesonide sprayed with MDI and swallowed has been successfully administered (104, 105) in a small series of pediatric cases showing a low systemic bioavailability and a good safety profile.

Viscous oral administration of budesonide (64, 106–108) achieves higher histological remission rates than when it is sprayed and swallowed, owing to a longer contact time with the esophageal mucosa. High doses of fluticasone have recently demonstrated better response rates in children and in adults (103).

About 25–40% of the patients treated with TCs fail to achieve histological remission. Nevertheless, there are promising preliminary data with new formulations of budesonide (viscous solution, effervescent tablets) specifically designed for EoE. They achieved 100% remission in just 2 weeks of treatment (108).

Predictors of response to corticosteroids are poorly understood (109, 110). Recent genomic analysis will probably help us in the identification of patients with better likelihood for treatment response.

We know about the safety of short-term TCs in EoE, but there are a few studies that discuss the long-term safety as well. Oropharyngeal cavity and esophageal candidiasis may occur in 10% of the treated patients, being usually asymptomatic (98, 104). Other studies indicate the possibility of suppression of the adrenal axis (111, 112). Cortisol monitoring to prevent adrenal insufficiency could be advisable for children receiving high doses of swallowed TCs for long periods or concomitant use of inhaled/nasal corticosteroids for associated atopic diseases.

The optimal long-term maintenance dose of TCs is yet to be determined. The maintenance treatment is recommended with the minimum effective dose to maintain clinical and histological remission (90, 113). The main drawback of corticosteroid treatment is that almost all patients relapse rapidly after cessation of therapy. Therefore, they are able to control the disease but not cure it.

A-2.1) Systemic corticosteroids: their efficacy is similar to that of swallowed corticosteroids, but the risks of side effects are higher. Their use is restricted to emergency situations with severe dysphagia or significant weight loss (7, 8, 102, 114, 115).

B) Elimination diet

The goal of dietary treatment strategies is to induce remission of EoE and, subsequently, help in the identification of food triggers, so as to avoid them and remain asymptomatic without the need for drugs.

Three possible strategies for dietary treatment have been described: elemental diets (ED), allergy testing-based elimination diets (ATBD), and empiric elimination of common dietary antigens that includes six-food elimination diet (SFED), four-food elimination diet (FFED), or two-food elimination diet (TFED) (116).

To evaluate the patient's response to an elimination diet, clinical, and histological remission must be achieved, and the only way to know this is by performing endoscopy with biopsy: firstly after eliminating and secondly after reintroducing the offending food (8). The initial elimination diet will be established for 6 weeks. If remission is achieved, the sequential reintroduction of the eliminated foods (one group every 6 weeks) is carried out, with clinical and histological controls for each group. The food identified as responsible for causing EoE will be eliminated from the diet permanently. This process involves performing multiple endoscopies, which is cumbersome, and the overall uptake amongst clinicians is unknown and should be limited because of the high costs and safety concerns as well as the associated inconvenience for the patient, particularly children. Patient's acceptance of this strategy improves if sedation is provided during the endoscopy and if they are performed in well-defined time frames after reintroduction of the food, avoiding dietary restrictions for a longer time than necessary (117). This is, obviously, necessary in children, in which case repeated deep sedation with propofol has been demonstrated to be safe (118).

To carry out an elimination diet, joint assessment with a nutritionist is mandatory to ensure an adequate caloric, electrolyte, and micronutrient intake (119), and it also helps in the assessment of dietary compliance in the patient. A multidisciplinary team is required, including an allergologist, for interpreting food labels, identifying possible sources of contamination with hidden allergens, and avoiding cross-reactivity of foods. The order of reintroduction of food can be adapted to the nutritional requirements or personal preferences of each patient.

B-1) Elemental diet

Elemental formulas based on essential amino acids lack proteins, thus, any allergenic source of the diet is eliminated. The carbohydrates and fats necessary to cover the daily dietary requirements are also added.

The initial efficacy data (120) have subsequently been supported by a long series of pediatric cases and adults, displaying remission results that exceed 90% (121–125). Despite its high efficacy, the application of elemental diet in clinical practice is very limited because of its high cost (and it not being universally covered by health insurances), poor palatability, lack of adherence, and the need for nasogastric tubes in most of the children. In addition, the process of reintroduction of food is longer and, therefore, requires a greater number of endoscopies. The elemental formulas can be used as a nutritional supplement in other types of diets (119).

B-2) Allergy testing-based food elimination diet (ATBD)

Spergel et al. (126) using a combination of prick test and atopy patch tests in children, described an efficacy of about 75% in the elimination diet based on the positive results obtained from these tests (126, 127). These results, that were methodologically incorrect, could not be reproduced later (123, 128–131). After 10 years, the same group published a broader series of studies with a remission rate of 53% (124). It is important to point out that food patch tests are not standardized. The same authors propose to include the elimination of milk, since this food gives a low negative predictive value (NPV) in the skin tests (124). A recent meta-analysis revealed that this dietary approach led to histologic remission of 45.5% (132), when compared with the 72% observed with SFED and the 90.8% with elemental diet.

A diet based study on the elimination of foods demonstrated an sIgE value ≥0.1kU/L and showed clinical-histological remission in 73% of the patients with fewer endoscopies than the SFED group. This study comprised of sIgE analysis for the six foods included in the group of SFED, and patients with negative sIgE were selected for the SFED (133). The elimination diet guided by blood IgE microarrays (component-resolved allergy diagnosis) has not been demonstrated to be effective as it had poor efficacy (7% histologic remission) (134).

B-3) Six-food group elimination diet (SFED)

The empirical elimination of the six-food group most commonly associated with food allergy (milk, wheat, soybean/legumes, egg, peanut/nuts, and fish/shellfish) achieved clinical-histological response in 72% of the patients. It has been proven to be highly reproducible in various studies in children and in adults (132, 135–138).

Milk and wheat are identified as the most common causes of EoE, followed by eggs and legumes/soybeans.

In a follow-up study after 1 year and 3 years, respectively, there was no clinical-pathological recurrence if the elimination of the identified foods was maintained (135, 139). A Spanish study (60) identified a single causative food in 71% of the adult patients (133). In another study series including North American children, it was 72% (139). The existence of several causative foods for a long-term avoidance diet complicates compliance. The differences detected in the causative food may be because of dietary habits and specific sensitization patterns in different geographical areas (140). Therefore, the elimination diets should be adapted to each country or region.

Several studies have analyzed less strict elimination diets, easier to comply, with reduced number of endoscopic procedures and shortened diagnostic process time.

Thus, the empiric FFED (milk, wheat, egg, and legumes including soy) proved its effectiveness in two prospective studies: the first in 54% of a group of adult Spanish patients (141) and the second in 64% of a group of children from the US (142). It consisted of eliminating, at first, the two most common food triggers (milk and cereals with gluten) and then increasing the level of restriction in non-responders.

Since milk is the most common food trigger in EoE, CM elimination diet has been proposed, achieving histological remission in 64% of children (143, 144). An extensive hydrolyzed formula of CM proteins has been well-tolerated in 88% of the adult patients with EoE caused by milk (145) as well as homemade or purchased products cooked with milk such as bread, muffins, or cakes for at least 6 weeks (146). This variety of milk products may improve compliance, quality of life, and nutrition in these patients. However, in a study that is being carried out by our group, 5 patients with EoE due to milk were allowed to have baked milk in their diet, and 6 weeks later all of them relapsed (unpublished data).

In summary, PPIs, topical steroids, or elimination diets might be offered as first line therapy for EoE patients. The question of which therapy would be administered first should be individually discussed with each patient and their relatives, and this might be interchangeable over time depending on the evolution of the disease and the patient's preferences. The efficacy of the therapy should be confirmed after 6–12 weeks with a follow-up endoscopy.

Endoscopic dilation is used for patients with severe dysphagia/food impaction with inadequate response to anti-inflammatory treatment. The complexity of EoE treatment regimens and frequent follow-ups require a multimodal, multidisciplinary management approach to optimize patient care (147).

C) Other treatments

Other treatments are also used for allergic diseases. Drugs such as montelukast and sodium cromoglycate are not recommended for the treatment of EoE, since neither of them showed any effects on symptom relief or in histological findings (148).

Various monoclonal antibodies have been investigated with unsatisfactory results.

Anti IL-5: Reslizumab treatment was studied in comparison with placebo in a study with 226 pediatric patients. The active treatment reduced the eosinophil count in the esophageal biopsy without significant symptom improvement (149). Two randomized controlled trials with mepolizumab have been reported in children and in adults (150), which showed no histological remission or differences in symptoms when compared with placebo.

Anti-TNF: Infliximab failed to reduce esophageal inflammation or to dismiss symptoms in a pilot study with three EoE patients (151).

Anti IgE: Omalizumab failed to reduce EoE-attributed symptoms or tissue eosinophil counts (152). In an open, non-blinded study in 15 patients, symptoms improved, but esophageal eosinophilia persisted after 3 months of treatment in 33% of the cases (153). A double-blind, placebo-controlled trial showed similar efficacy with placebo (154).

Anti CRTH2: CRTH2 is a G protein-coupled receptor expressed in all the cells that are involved in Th2 inflammation. OC000459 acts as an oral bioavailable CRTH2 antagonist. In a randomized, double-blind, placebo-controlled study with 26 patients diagnosed with EoE (155), eosinophil counts in the esophagus were significantly reduced in 36% of the patients. At the same time, the clinical assessment improved significantly in the active group but not in the placebo one.

It is important to point out the importance of IL-4, IL-13, and their receptors in allergic diseases, with an emphasis on asthma, atopic dermatitis, and EoE (156). Therefore, the following agents are being investigated.

Anti IL-13: A recent trial with QAX576 (157) showed a tendency to improve symptoms with a reduction of eosinophil levels in the esophagus (60% decrease vs. an increase of 23% with placebo).

Anti IL-4: A randomized controlled trial with dupilumab is ongoing in adults with moderate or severe active EoE (NCT02379052 www.clinicaltrials.gov) (158).

Anti TGF-β: An open trial with losartan, an angiotensin II receptor blocker that inhibits TGF-β1, is underway in patients with EoE (NCT0180816 www.clinicaltrials.gov) (158).

Eotaxin anti-CCR3 receptor: An oral CCR3 receptor blocker is currently under development (158).

Azathioprine: A series of studies conducted in three adults with severe and cortico-dependent EoE was published. After treatment with azathioprine, they showed a symptomatic and histological response, relapsing when treatment was interrupted. Possible side effects limited the use of this drug (159).

A better understanding of the phenotypes and endotypes of allergic diseases has resulted in rapid development of biologic medications that target multiple steps of the inflammatory pathways. All of the biologic medications currently approved and most of the biologic medications under development for allergic diseases have focused on the Th2 inflammatory pathway. The novel biologic therapies that have emerged over several years in the past have amazingly improved the management of patients with refractory allergic disease as well as with EoE (160).

D) Endoscopic dilation

Mechanical dilation of the esophagus is useful when there is an established subepithelial fibrosis that causes esophageal narrowing. The goal is to relieve dysphagia and to achieve an esophageal caliber suitable for the proper passage of food. This treatment is effective in 75% of the patients (71), since the symptoms improve immediately. However, it cannot be considered as a first-line treatment or as a monotherapy, as it has no effect on the underlying inflammatory process. It should be considered in patients with dysphagia or esophageal narrowing and in non-responders to anti-inflammatory treatment. Esophageal dilation has always been associated with risks in EoE. However, two systematic reviews demonstrated that the risk of esophageal perforation occurred in less than 1% of the cases, which is similar to the risk involved in dilating esophageal stenosis caused by other etiologies (161, 162).

## Conclusion and future perspectives

Eosinophilic esophagitis has been known as a distinct disease entity only for the past two decades, but the progress that has been made by stakeholders regarding the understanding of EoE's pathogenesis, genetic background, natural history, allergy workup, standardization in assessment of disease activity, evaluation of minimally invasive diagnostic tools, and new therapeutic approaches is enormous (163). The incidence and prevalence of EoE have also increased in the same way, being the leading cause of food impaction, the major cause of dysphagia, and accounting for high health-related costs. Therefore, in a few years, EoE may no longer be considered as a rare disease.

The main novelty of the new guidelines on EoE (9) is the retraction of the term “PPI-REE,” not considering the response to PPIs as a diagnostic criterion anymore. In the treatment of EoE, PPIs are now at the same level as diets or topical steroids, and the choice of therapy should be individually discussed with the patients and relatives.

The multistage step-up elimination diet management approach of EoE is one of the most important solutions in the treatment of this disease (9), because its advantages may definitely help in the improvement of adherence to dietary therapy not only in EoE patients and their families but also in health professionals and caregivers.

Further studies are needed the development of birth cohort studies, non-invasive disease monitoring methods, biomarkers for routine practice, the development of new therapies, comprehensive standardized scoring systems for symptoms, quality of life, increase in awareness about EoE, novel food allergy testing to detect triggering foods in EoE and the implications of cross reactivity of food allergens, drug and doses required for initial therapy and safety issues with maintenance therapy (especially in children).

Multidisciplinary management of EoE, involving gastroenterologists, pediatricians, allergists, pathologists, dietitians, and ENT specialists, is required. The recent formation of the Consortium of Eosinophilic Gastrointestinal Disease Researchers (part of the Rare Disease Clinical Research Network of the National Institutes of Health) and EoE Connect will help to improve the understanding and treatment of EoE (https://www.rarediseasesnetwork.org/cms/cegir/ and https://eoeconnect.eu/).

## Author contributions

All authors listed have made a substantial, direct and intellectual contribution to the work, and approved it for publication.

### Conflict of interest statement

The authors declare that the research was conducted in the absence of any commercial or financial relationships that could be construed as a potential conflict of interest.
